# The time course of the spatial representation of ‘past’ and ‘future’ concepts: New evidence from the STEARC effect

**DOI:** 10.3758/s13414-024-02862-1

**Published:** 2024-02-27

**Authors:** Gabriele Scozia, Mario Pinto, Silvana Lozito, Nicola Binetti, Mariella Pazzaglia, Stefano Lasaponara, Fabrizio Doricchi

**Affiliations:** 1 Dipartimento di Psicologia, Università degli Studi di Roma ‘La Sapienza’, Roma, Italy; 2 Fondazione Santa Lucia IRCCS, Roma, Italy; 3 International School for Advanced Studies (SISSA), Trieste, Italy

**Keywords:** Time, STEARC effect, Space-time association, Mental time line

## Abstract

Humans use space to think of and communicate the flow of time. This spatial representation of time is influenced by cultural habits so that in left-to-right reading cultures, short durations and past events are mentally positioned to the left of long durations and future events. The STEARC effect (Space Temporal Association of Response Codes) shows a faster classification of short durations/past events with responses on the left side of space and of long durations/future events with responses on the right side. We have recently showed that during the classification of time durations, space is a late heuristic of time because in this case, the STEARC appears only when manual responses are slow, not when they are fast. Here, we wished to extend this observation to the semantic classification of words as referring to the ‘past’ or the ‘future’. We hypothesised that the semantic processing of ‘past’ and ‘future’ concepts would have engaged slower decision processes than the classification of short versus long time durations. According to dual-route models of conflict tasks, if the task-dependent classification/decision process were to proceed relatively slowly, then the effects of direct activation of culturally preferred links between stimulus and response (S-R), i.e., past/left and future/right in the case of the present task, should attain higher amplitudes before the instruction-dependent correct response is selected. This would imply that, at variance with the faster classification of time durations, during the slower semantic classification of time concepts, in incongruent trials, the direct activation of culturally preferred S-R links should introduce significant reaction time (RT) costs and a corresponding STEARC at the fastest manual responses in the experiment too. The study's results confirmed this hypothesis and showed that in the classification of temporal words, the STEARC also increased as a function of the length of RTs. Taken together, the results from sensory duration and semantic classification STEARC tasks show that the occurrence, strength and time course of the STEARC varies significantly as a function of the speed and level of cognitive processing required in the task.

**Supplementary Information:**

The online version contains supplementary material available at 10.3758/s13414-024-02862-1.

## Introduction

Humans from different languages and cultures frequently use spatial metaphors to represent the flow of time. Some of these representations are derived from sensorimotor experiences linked to cultural scanning and reading habits. For example, in left-to-right reading cultures, time flows from left to right, so that, ‘short’ sensory durations and past events are placed on the left, while ‘long’ durations and future events are on the right side of mental space. Other spatial representations of time are derived from culture-independent sensorimotor habits like forward locomotion, so in this case, the ‘past is left behind’ and the future projected ‘ahead’ (Bonato et al., 2012; Boroditsky, 2001; Casasanto & Bottini, 2014; Casasanto and Boroditsky, 2008; Lakoff & Johnson, 1999; Núñez & Sweetser, 2006; Tversky et al., 1991). Some of the most compelling experimental evidence of spatial representation is seen in the STEARC effect (Spatial Temporal Association of Response Codes). The STEARC consists of faster classifications of short time durations with manual responses on the left side and long durations with responses on the right side of space, rather than vice versa (Conson et al., 2008; Ishihara et al., 2008; Vallesi et al., 2008).

In a recent study (Scozia et al., 2023a) we investigated whether, during the classification of the ‘short’ versus ‘long’ durations of simple visual stimuli, the presence of the STEARC and its strength vary as a function of the speed of reaction times (RTs). To this aim, we divided the distribution of RTs into four proportional quartile bins, from slowest to fastest RTs, and examined the time course of the STEARC across these bins. Surprisingly, we found that the STEARC emerges only when decisions on time durations are slow, i.e. at late bins, while no STEARC is present with fast decisions at early bins. These results suggest that activating a left-to-right mental spatial representation of time flow is a relatively late event that superimposes on an earlier and faster non-spatial representation of time. The same finding shows that it is empirically possible to separate two co-existing mechanisms of time coding: a fast non-spatial mechanism and a slow spatial one.

Interestingly, the STEARC is also observed at a higher level of conceptual-semantic processing, for example, during the classification of words and sentences as referring to the ‘past’ or the ‘future’ (Santiago et al., 2007; Torralbo et al., 2006). This finding also extends to pictorial stimuli. For example, Santiago et al. (2010) showed that following the presentation of a sequence of six pictures or a video depicting a short story, observers are faster at classifying ‘early’ pictures or frames in the story with a left-side response button and ‘late’ pictures or frames with the right-side button.

The retrospective analysis of available evidence suggests that the higher-level semantic processing of temporal words, phrases or pictures (Santiago et al., 2007; Santiago et al., 2010; Torralbo et al., 2006) entails much slower RTs as compared to the classification of the ‘short’ versus ‘long’ sensory duration of visual or acoustic stimuli (Scozia et al., 2023a; Vallesi et al., 2008; see Study 2 in the present report for an empirical test with our data). On this ground, we wanted to extend our previous investigation of the time course of the STEARC from the classification of time durations (Scozia et al., 2023a) to the semantic classification of words as referring to the ‘past’ or the ‘future’ (Santiago et al., 2007). At variance with simple judgements of durations that at fast RTs exploit non-spatial sensorimotor mechanisms of time processing, we expected that using semantic concepts like ‘past’ and ‘future’ in the classification words would have engaged more complex and slow cognitive mechanisms. In this regard, dual-process models of conflict task (Kornblum et al., 1990; Miller & Schwarz, 2021; Ridderinkhof, 2002) make rather precise predictions on the relationship between stimulus-response (S-R) correspondence effects and the speed of processing of target-relevant features. In particular, the ‘Activation-Suppression hypothesis’ proposed by Ridderinkhof (2002) holds that the direct activation of the response resulting from irrelevant target features is selectively suppressed in favour of the instructed association between the target-relevant feature and the motor response, and that this suppression needs time to build up. One of the predictions derived from the ‘Activation-Suppression hypothesis’ is that if deliberate, i.e., instruction-dependent, decision processes were to proceed relatively slowly, the activation of incorrect responses along the instruction-independent route would attain higher amplitudes before the activation of the correct response along the deliberate instruction-dependent route is attained. In the case of STEARC tasks requiring the semantic classification of temporal words, this would imply that in incongruent trials, the activation of culturally preferred left/past and right/future associations would attain higher amplitudes before the deliberate and task-dependent activation of opposite S-R mappings, i.e., left/future and right/past, is reached for the selection of the correct response. Therefore, in this case, and at variance with the discrimination of sensory time durations, the presence of the STEARC should be expected even at the fastest RTs.

Here, in Study 1, we tested this prediction, and then, to substantiate the premise that slower RTs would have been observed during the classification of temporal words as compared with the classification of sensory time duration, in Study 2, we ran a direct comparison between the results of the present study and those from our previous STEARC study with time durations (Scozia et al., 2023a).

## Study 1

### General methods

The study was designed following the principles of the Declaration of Helsinki and was approved by the Ethics Committee of the Department of Psychology – Sapienza University of Rome (Protocol Number: 0002619).

### Participants

To determine the number of participants, we ran an a priori power analysis (G*Power program; Faul et al., 2007) using the effect size f(U) = 0.439 derived from the previous study of Santiago et al. (2007). This analysis showed that 27 participants would be needed to have a power of .90, considering an alpha of .05 (two-sided) of statistical significance for a repeated-measures within-factors ANOVA. Based on this preliminary analysis, we tested 35 healthy adult participants. All participants (24 female and 11 male, mean age = 24.02 years, SD = 3.92) were naïve, right-handed and non-bilingual Italian native speakers.

### Apparatus

Due to the COVID pandemic restrictions, experiments were administered through the open-source software OpenSesame (https://osdoc.cogsci.nl/3.3/; Mathôt et al., 2012), imported on a Jatos Server (https://www.jatos.org/). Participants accessed the experiment using a General-Multiple Worker Link. Participants were instructed to run the experiment in a quiet and isolated room and wear in-ear plug headphones to reduce environmental noise sources. They were also asked to keep their head positioned at a viewing distance of 60 cm from the screen. All participants had normal or corrected-to-normal vision and were naïve to the aim of the study. Instructions for all experiments were provided during individual audio/video calls with one of the experimenters. A training block that included 24 trials was administered before the experimental session and always corresponded to a shortened version of the first experimental block.

### Procedure and stimuli

**Fig. 1:**
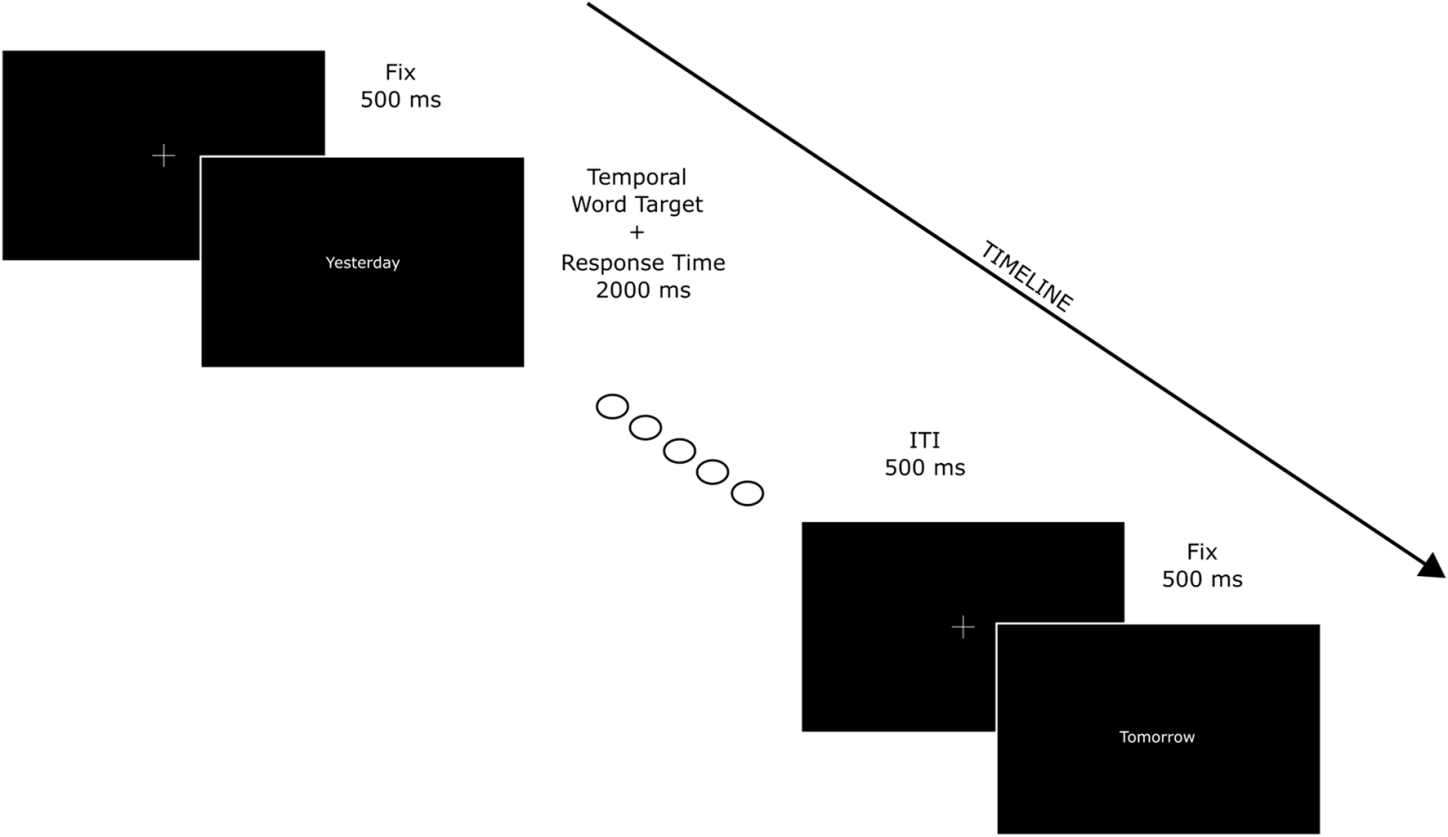
Examples of two consecutive trials. The first is a temporal trial with the past word ‘Yesterday’, while the second is a temporal trial with the future word ‘Tomorrow’

Each trial started with the 500-ms presentation of a central fixation cross (1.5° × 1.5°). At the end of this delay, a linguistic temporal target (verbs and adverbs explicitly referred to the past or future) replaced the central fixation cross. Target stimuli remained available for response for 2,000 ms (Fig. 1). Participants were asked to respond by using one out of two response buttons on the keyboard: one on the left (x) and one on the right side of the keyboard (m).

Participants performed a STEARC task in two different experimental conditions: (a) in the ‘Congruent’ condition, they were asked to respond to past words with the left hand/button and future words with the right hand/button; (b) vice versa in the ‘Incongruent’ condition, they were asked to respond to past words with the right hand/button and future words with the left hand/button. Participants were asked to respond as quickly as possible. Twenty past and 20 future words served as target stimuli (see word list in the Online Supplementary Material). According to the Italian normative data, past and future words were matched for length and frequency of use (p > 0.4, http://143.50.35.46/it/cerca). Each block/condition consisted of 40 past and 40 future trials (two repetitions per word). The administration order of the Congruent and Incongruent conditions was counterbalanced among participants. Following the method adopted in previous studies (Santiago et al., 2007), RTs shorter than 250 ms and longer than 2,500 ms were excluded from the analyses.

### Statistical analyses

The STEARC effect was estimated as a function of the decreasing speed of RTs ranked along four quartile bins (Rubichi et al., 1997). To examine the temporal dynamics of the STEARC, we used the Vincentization procedure introduced by Ratcliff (1979; see Pinto et al., 2021a, 2021b; Rubichi et al., 1997; Scozia et al., 2023a). For each participant, we calculated the RT distributions of correct responses, i.e., from fastest to slowest, in Congruent and Incongruent trials. We then divided each distribution into four proportional quartile bins so that each bin contained the same proportion of trials, i.e., one-fourth. The difference between mean RTs from corresponding bins in Congruent and Incongruent trials is a bin-by-bin measure of the time course of the STEARC. Finally, individual RTs were entered in a Congruency (Congruent vs. Incongruent) × Temporal Words (Past vs. Future) × RTs-Bin (Bin1 vs. Bin2 vs. Bin3 vs. Bin4) repeated-measures ANOVA. In addition, we ran separate ANOVAs for ‘past’ and ‘future’ words.

### Results

**Fig. 2:**
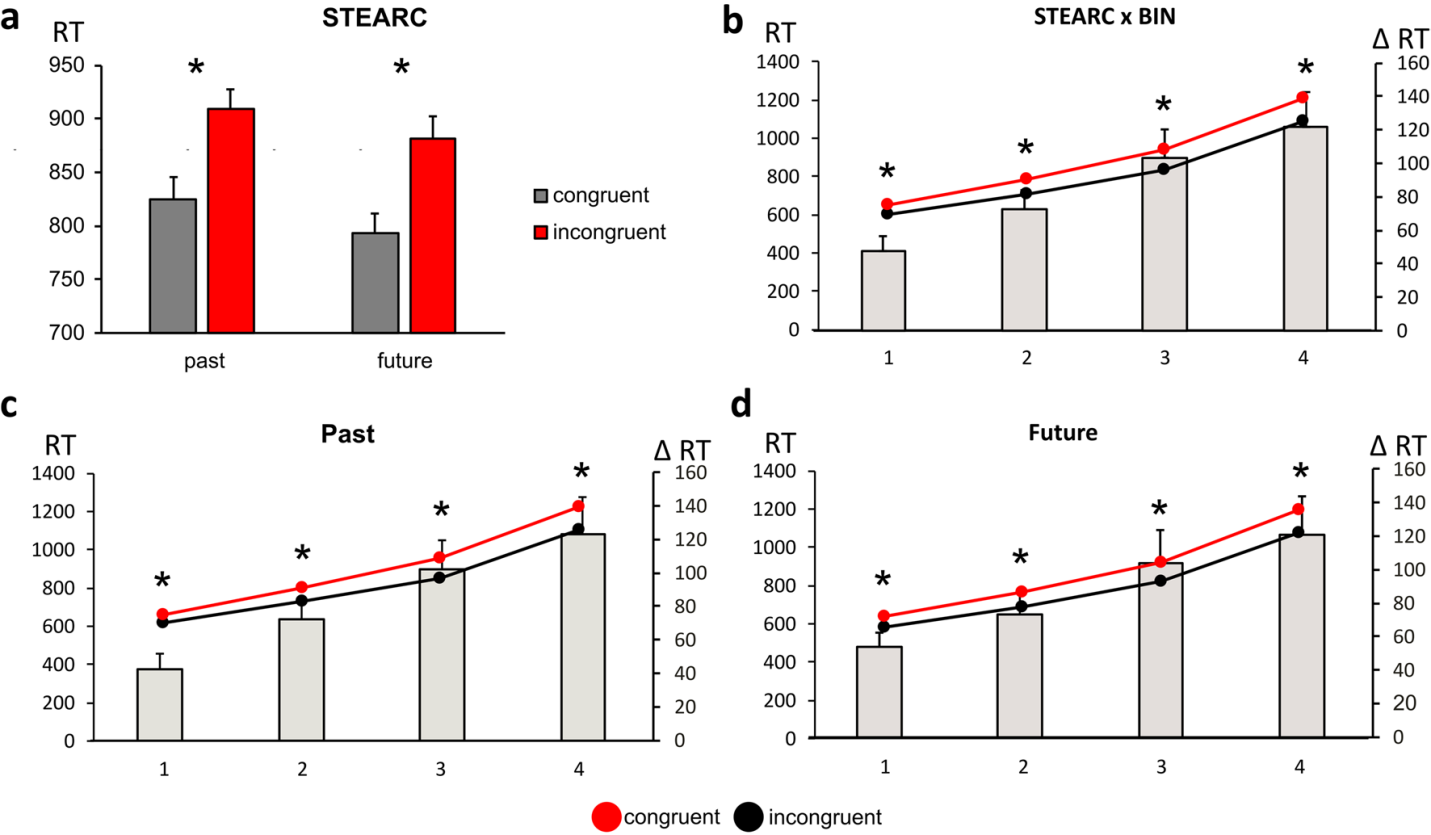
(**a)**: Average reaction times (RTs) to Past and Future words in the Congruent and Incongruent conditions. (**b)**: STEARC (Spatial Temporal Association of Response Codes) effects as a function of the speed of RTs: Bin1 has the fastest RTs, and Bin4 has the slowest RTs. Left Y-axis: RTs in the Congruent (black line) and Incongruent (red line) conditions. Right Y-axis: Incongruent minus Congruent RTs difference (grey bars). **(c)**: STEARC effects with ‘Past’ words as a function of the speed of RTs: Bin1 has the fastest RTs, and Bin4 has the slowest RTs. Left Y-axis: RTs in the Congruent (black line) and Incongruent (red line) conditions. Right Y-axis: Incongruent minus Congruent RTs difference (grey bars) for past and future word targets. **(d)**: STEARC effects with ‘Future’ words. Significant differences are indicated with an asterisk

The ANOVA highlighted a significant main effect of Congruency [F (1,34) = 49.702, p < .001, η_p_^2^ = .593], with faster RTs to Congruent (808 ms) than to Incongruent targets (895 ms). The Congruency × Temporal Words interaction was not significant [F (1, 34) = .060, p = .807, η_p_^2^ = .001; see Fig. 2a]. A significant Congruency × RTs-bin interaction [F (3, 102) = 15.735, p < 0.001, η_p_^2^ = 0.316] showed that the STEARC was significant at each bin and that its strength increased as a function of decreasing speed of RTs along the four consecutive bins (all Bonferroni post hoc p < 0.001; see Fig. 2b). The Congruency × Temporal Words × RTs-bin interaction was not significant [F (3, 102) = 0.212, p = .887, η_p_^2^ = .006; see Fig. 2c]. A significant main effect of Temporal Words [F (1,34) = 12.396, p = .001, η_p_^2^ = .267] indicated faster RTs for future (837ms) than past words (866). With both past and future words, the significant Congruency × RTs bin interaction [F (1,34) = 11.486, p < .001, η_p_^2^ = .252 for past words; F (1,34) = 8.392, p < .001, η_p_^2^ = .197] showed an increase in the STEARC when moving from the fastest to the slowest responses.

## Study 2

### Comparison between the speed of classification of ‘past/future’ words and the speed of classification of ‘short/long’ time durations in STEARC tasks

Here, we wished to make a direct comparison between the RTs recorded in the present STEARC experiment (N = 35) and those recorded in a previous equivalent STEARC task that required the classifications of 1-s ‘short’ versus 3-s ‘long’ durations of simple visual dot stimuli (Scozia et al., 2023a, 2023b; N = 25). Participants in the two experiments were all right-handed university students. The two groups did not differ in age (Durations: mean age = 23.40 years, SD = 2.76; Words: mean age = 24.02 years, SD = 3.92; p = .49) or gender composition (Durations: 17 female and nine male; Words: 24 female and 11 male; p = .71).

**Fig. 3:**
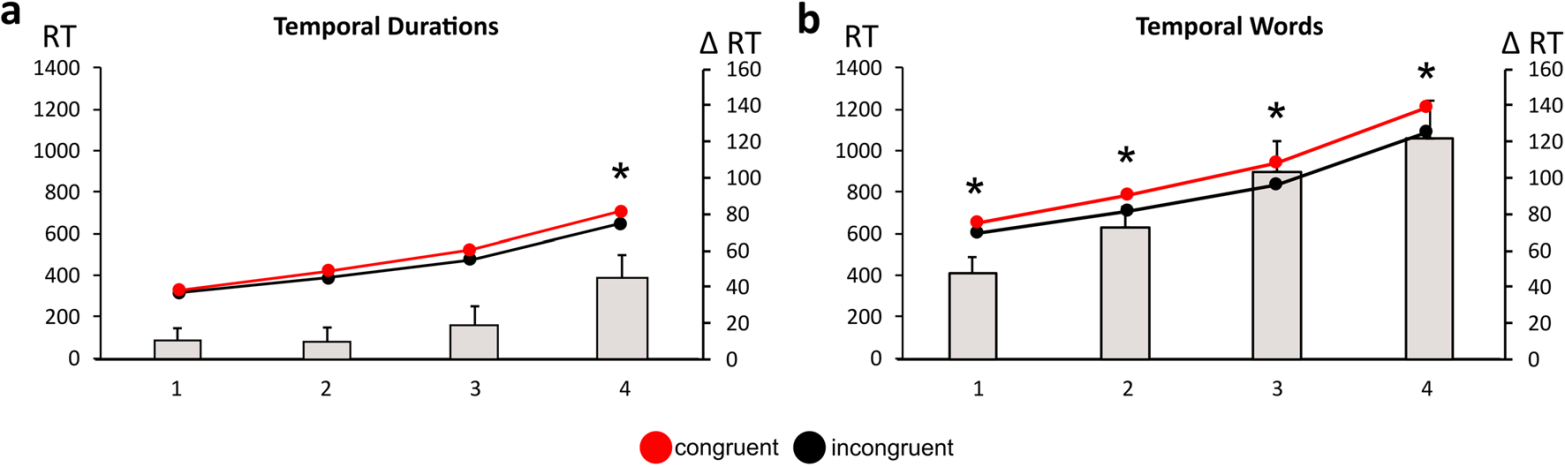
STEARC (Spatial Temporal Association of Response Codes) effects as a function of the speed of reaction times (RTs): Bin1 has the fastest RTs, and Bin4 has the slowest RTs. Left Y-axis: RTs in the Congruent (black line) and Incongruent (red line) conditions. Right Y-axis: Incongruent minus Congruent RTs difference (grey bars) obtained (**a**) data from the temporal duration STEARC task (Scozia et al., 2023a) and (**b**) data from the present temporal words classification STEARC task. Significant differences are indicated with an asterisk

Individual RTs were entered in a Task (Temporal Words, Temporal Durations) × Congruency (Congruent, Incongruent) × RTs-Bin (Bin1 vs. Bin2 vs. Bin3 vs. Bin4) mixed ANOVA. RTs were slower with Temporal Words than with Temporal Durations [F (1,58) = 282, p < .0001, η_p_^2^ = .82; temporal words: 852 ms, time durations: 407 ms; see Fig. 3]. The ANOVA also highlighted main Congruency [F (1,58) = 44, p < .0001, η_p_^2^ = .43] and RTs-Bin [F (3,174) = 885, p < .0001, η_p_^2^ = .93] effects. A significant Task × Congruency × RTs-Bin interaction [F (3,174) = 3.7, p = .01, η_p_^2^ = .08] originated from the fact that a Bonferroni post hoc comparison showed that with Temporal Durations, the STEARC was only found at the slowest RTs in bin 4 (p < .001), confirming findings from Scozia et al. (2023a). In contrast, with Temporal Words, the STEARC was present at each RTs Bin (all p < .001).

## Discussion

The main purpose of the present study was to investigate whether, during the semantic classification of words as referring to the ‘past’ or the ‘future’, the significance and strength of the STEARC vary as a function of the speed of RTs. The results show that when cognitive resources are engaged in this semantic classification, the STEARC is found at all RT bins from fastest to slowest responses. On the one hand, this is at variance with the STEARC that is found during the classification of the duration of simple visual stimuli, where the STEARC is absent with fast responses (Scozia et al., 2023a), a finding showing that, in that case, the activation of a nurtured left-to-right spatial representation of durations is a late cognitive heuristic that gets superimposed on an earlier non-spatial coding of time durations. On the other hand, in both the case of time durations and the semantic classification of ‘past’ versus ‘future’ words, the strength of the STEARC increased as a function of RTs length, a trend that is opposite to that found in many visual Simon tasks (Craft & Simon, 1970; Miller & Schwars, 2021; Simon, 1990), where congruency effects are stronger at fast RTs and progressively decay and even reverse at longer RTs (De Jong et al., 1994; Rubichi et al., 1997). The increasing size of the STEARC as a function of RT length found in time duration and semantic temporal classification tasks suggests that the activation of a spatial representation of time that is directionally congruent with acquired scanning and reading habits is an incremental phenomenon. The non-fully immediate implementation of the spatial representation of time that is found in time-duration tasks seems in agreement with the idea that the spatial representation of time is characterized by the existence of alternative and flexible mappings that are available to the same individual or individuals with different language and cultural conventions (Boroditsky, 2001; Lakoff & Johnson 1999). More generally, our results support the hypotheses that people can conceptualize the temporal domain and the different features of the same domain, for example, duration versus temporal concepts, in entirely different ways (Santiago et al., 2011) and depending on the type of cognitive processing required by the task.

The increasing size of the STEARC as a function of RT length is in agreement with evidence in the domain of the Space-Number association. This shows that the strength of the SNARC effect, i.e., faster left-side responses to small numbers than to large numbers and faster right-side responses to large numbers than to small numbers (Dehaene et al., 1993), grows as a function of RT length (Didino et al., 2019; Gevers et al., 2006; Yan et al., 2021). This phenomenon was explained by the dual-route model (Gevers et al., 2006): since both the unconditional route (i.e., an automatic, long-term space-number mapping) and a conditional route (i.e., an arbitrary task-specific mapping) are always simultaneously activated, the interference of the unconditional route increases along with response latency, inducing a stronger SNARC effect for longer RTs.

We sketch two possibilities to explain why, with duration judgements, the STEARC is only present at slow RTs, while during semantic temporal judgements, the STEARC is already present at the fastest RTs. With simple duration judgements, one might argue that time is not mapped into space at fast RTs, or that, according to the dual-route activation suppression framework proposed by Ridderinkhoff (2002; see also Miller & Schwarz 2021), in incongruent trials, the inhibition of a direct default route linking short durations to left-side responses and long durations to right-side responses is rapidly and effectively triggered in a large number of trials, thus producing no significant delay in response to incongruent trials at fast RTs. In contrast to this, one might speculate that during the cognitively demanding semantic classification of temporal words, which entails much longer RTs for the classification of time durations, target-relevant attributes are accessed so slowly that the activations of the unconditional-instruction independent route and the conditional instruction-dependent route overlap in the large majority of trials: this causes the STEARC to emerge even at faster RTs. These conclusions are in agreement with predictions from the ‘Activation-Suppressionn’ hypothesis (Ridderinkhof, 2002) that ‘ *the slower the processing in the deliberate* task-dependent *decision route*, *the more time there is for response activation along the direct* unconditional *route*’, so that slower responses are present in incongruent trials. Nonetheless, it is important to rememeber that in tasks like the STEARC, there is no irrelevant stimulus feature, for example, left- or right-side location of the target, that triggers the unconditional S-R association: instead, the activation of culturally preferred associations, for example, small-left or short/past-left, and task-defined and non-culturally preferred associations, for example, small-right or short/past-right, are both contingent upon the discrimination of the target-relevant feature.

Here, it is also important to note that in dual-route models of conflict S-R tasks, ‘*direct activation effects are unconditional, in the sense that the response activated via the direct route is independent of S-R mapping instructions*’ (Ridderinkhof, 2002), so that, for example, a left-side stimulus will always and automatically activate a left-side response. Similar unconditional and automatic mappings are also considered to be in action in cognitive tasks with no lateralised stimuli like the SNARC task, where small numbers are linked ‘by default’ to left-side responses and large numbers to right-side ones (Gevers et al., 2006), or the STEARC task where short durations are considered to be linked ‘by default’ to left side responses and long durations to right-side ones (Conson et al., 2008; Vallesi et al., 2008). Nonetheless, recent empirical evidence points to the qualification and reformulation of these assumptions, in both the numerical and the temporal domains. First, contrary to the assumption that number magnitude has an inherent spatial representation (Fischer et al., 2003), several investigations now suggest that the use of contrasting left/right spatial codes plays a fundamental role in the generation of the mental left-to-right spatial organisation of numbers (Aiello et al., 2012; Fattorini et al., 2015; Pinto et al., 2018; Pinto et al., 2019a; Pinto et al., 2019b; Pinto et al., 2021a; Pinto et al., 2021b). This organisation occurs both when the conceptual contrast between left/right spatial codes regulates the selection of a spatially defined motor response, like in the SNARC task, and when left/right spatial codes are jointly activated together with small/large number magnitude codes in the instruction that regulates the selection of a non-spatial Go versus No-Go non-spatial response, like in the Implicit Association Task (Fischer & Shaki, 2017; Nosek & Banaji, 2001; Pinto et al., 2019a; Pinto et al., 2021a). These findings suggest that the conceptual left/right contrast on which response codes are mapped induces the re-activation of left-to-right reading habits acquired to inspect and order the ascending series of number magnitudes.

Second, following these results from the number domain, it could be argued that no spatially organised Mental Time Line (MTL) is evoked when left/right spatial codes are not jointly activated with short/long duration or past/future temporal codes for response selection. Past evidence suggests that the task relevance of the temporal dimension has a crucial impact in triggering the spatial representation of time (von Sobbe et al., 2019), and that the left-to-right spatial coding of time is not automatic and could rather be dependent on the use of spatial and temporal response codes in the task at hand (Anelli et al., 2018; Ulrich & Maienborn, 2010; Weger & Pratt, 2008). We provided evidence supporting this conclusion in a recent series of four experiments (Scozia et al., 2023b). We showed that in a uni-manual ‘Implicit Association Task’ (IAT; Nosek & Banaji; 2001), attending selectively to ‘past’ or to ‘future’ words does not activate corresponding ‘left’ or ‘right’ spatial concepts and, vice versa, attending selectively to ‘left’ or ‘right’ spatial concepts does not activate corresponding ‘past’ or ‘future’ temporal ones. These results strongly suggest that time has no inherent spatial representation, and that stable and reliable MTLs are rather triggered by the use of contrasting spatial ‘left/right’ or ‘backward/forward’ codes for response selection. Additional studies are required to further explore and draw comprehensive conclusions on this issue.

To conclude, together with the results of a previous study (Scozia et al., 2023a), the results from the present experimental report detail the time development of the STEARC effect as a function of the cognitive complexity of the temporal task performed by participants. S-R correspondence models can provide insight into the mechanism that likely subtends the temporal development of the STEARC. Nonetheless, time-resolved EEG or MEG investigations are necessary to fully clarify the neural and functional correlates of the behavioural phenomena we witnessed in our studies.

### Supplementary Information

Below is the link to the electronic supplementary material.

Supplementary file1 (DOCX 29 KB)

## Data Availability

The datasets generated and/or analysed during the current study are available from the corresponding author upon reasonable request.
