# Supplementary material for: The time course of the spatial representation of ‘past’ and ‘future’ concepts: New evidence from the STEARC effect
Source: Atten Percept Psychophys. 2024 Feb 27;86(3):1048–55. doi: 10.3758/s13414-024-02862-1 (PMC11062999; doi:10.3758/s13414-024-02862-1)
Supplement: Supplementary file 1 — Supplementary file1 (DOCX 29 KB) [file 13414_2024_2862_MOESM1_ESM.docx]

**Supplementary Material**

**The time course of the spatial representation of “past” and “future” concepts: new evidence from the STEARC effect**

*Gabriele Scozia ^1,3*^, Mario Pinto ^1,2^, Silvana Lozito ^1,2^, Nicola Binetti, Mariella Pazzaglia^1,2^, Stefano Lasaponara ^1,2^, and Fabrizio Doricchi^1,2 *^*

*^1^ Dipartimento di Psicologia, Università degli Studi di Roma ‘La Sapienza’, Roma, Italy*

*^2^ Fondazione Santa Lucia IRCCS, Roma, Italy, ^3^ International School for Advanced Studies (SISSA), Trieste, Italy*

*^*^Corresponding authors:* [*fabrizio.doricchi@uniroma1.it*](mailto:fabrizio.doricchi@uniroma1.it)*,* [*gabriele.scozia@uniroma1.it*](mailto:gabriele.scozia@uniroma1.it)

**List of past and future words**

| **Past** | **Future** |
| --- | --- |
| Passato | Futuro |
| Prima | Dopo |
| Recentemente | Prossimamente |
| Ieri | Domani |
| Disse | Dirà |
| Ebbe | Avrà |
| Parlai | Parlerò |
| Potemmo | Potremo |
| Cercaste | Cercherete |
| Provaste | Proverete |
| Andasti | Andrai |
| Apparii | Apparirò |
| Vide | Vedrà |
| Pensai | Penserò |
| Guardai | Guarderò |
| Chiese | Chiederà |
| Decideste | Deciderete |
| Facesti | Farai |
| Credesti | Crederai |
| Guidammo | Guiderai |
